# Syphilis, Pseudo-Syphilis, Mercury

**Published:** 1821-12-01

**Authors:** 


I. *The Scourge of Venus and Mercury, represented in a Treatise of the Venereal Disease; giving a succinct Account of the Nature, Causes, Signs, Degrees, and Symptoms of that dreadful Distemper; and the fatal Consequences arising from Mercurial Cures, with the several Ways of taking that Infection; of the virulent Gonorrhœa, the Caruncula or Excrescences in the Urinal Passage, the Phymosis and Paraphymosis, the Tumours of the Scrotum and Testicles, the Venereal Bubo, Warts, &c. With the other Symptoms, that are either antecedent, concomitant, or consequent, to the most inveterate Cases relating to that Distemper, such as Gleets, putrid Ulcers, &c. and their Cure, without one Grain of Mercury, in a plain and easy Method, founded upon unquestionable Experience of above Fifty Years. Unto which is added, the true Way of Curing, not only the consummate and inveterate, but also the mercurial Pox, found to be more*

*dangerous than the Pox itself. The whole illustrated by many authentic and unquestionable Accounts of Cures performed, after the Patients were reduced to the very Brink of the Grave, by mercurial Operations, the like not as yet extant.* By J. SINTELAER, Practitioner in Physic. London, 1709.

II. *Observations on Syphilis, principally with Reference to the Use of Mercury in that Disease.* By JOHN BACOT, Member of the Royal College of Surgeons, and late Surgeon of the Grenadier Regiment of Guards. One vol. 8vo, pp. 115. London, 1821.

III. *An Inquiry into the Effects of Mercury on the Human Body, with a View to estimate its Value as a Remedy in several important Diseases.* By HENRY THOMSON, M.D. Licentiate of the Royal College of Physicians, Physician to the Dispensary for Diseases of the Skin, &c. &c. Manuscript, quarto, pp. 56.

IV. *A comprehensive Treatise upon the Symptoms, Consequences, Nature, and Treatment of Venereal or Syphilitic Diseases.* Translated from the *Seventh* French Edition of F. SWEDIAUR, M.D. Two volumes in one, 884 pages, 8vo, 1820.

V. *Engravings of Diseased Appearances, taken from Drawings, with the Symptoms, Progress, and Treatment of the Cases.* By JOHN HARRISON, Member of the Royal College of Surgeons, and Assistant Surgeon to the First or Grenadier Regiment of Foot Guards. Quarto, three plates, coloured, with descriptive letter-press. London, 1821.

IT is by no means improbable that in the course of a century hence, some *flash* publication of the present day may, after a profound dive in the ocean of oblivion, be dragged up by some curious critic, and, like "the scourge of Venus and Mercury," at the head of this article, be exhibited (no doubt to its astonishment) in company one hundred years younger than itself.* If Dr. Sintelaer were to rise from his grave, with the tremendous wig and cloak in which he is depicted in the book before us, he would probably be more surprized at the novelty of our *dandy* costume, than at that of our modern doctrines on syphilis. Yet we can-

* It was this book which first excited Dr. Thomson (of Edinburgh) to the investigations which have since made so much noise in the world.

not but think that both the dress and address of medical men of the present day are improved since the time of Sintelaer, who dates his book in the genuine style of those worthies who issue their proclamations from Stanhope Street, Charlotte Street, Nelson Square, the Egyptian Hall, and Bolton Row—namely—"from my house in High Holborn, the late dwelling-house of His Grace the Duke of Leeds, *over against Little Turnstile.*" It is not a little remarkable, too, that this "Scourge of Venus and Mercury," is printed by G. Harris—"next door to the *Bagnio* in St. James' Street," and is embellished with a large plate, entitled, "The Martyrdom of Mercury," representing men and women with demolished noses, rotten tibiæ, exfoliated skulls, empty alveolæ, panther-like skins, fallen palates, and other attributes which the moderns, as well as Dr. Sintelaer, have liberally bestowed on that vile poison—MERCURY.

Dr. Sintelaer moralizes in "good set terms" on the folly of mankind in treading the path of vice, and running after the forbidden fruits of Venus, when they see such vast multitudes of those miserable objects, "those spectacles of horror and amazement returning on the same road."

> "But what seems to be more amazing still, is, that after these unfortunate creatures see themselves thus infected, by the *poisonous fruits Venus* bestows upon those, that with an uncontrouled desire will intrude themselves into her labyrinths, they should have this additional misfortune, to be entangled also in the most deceitful snares of Mercury, whose lash being so severe, as not only to tear the flesh, but also to strike to the very bones, their case instead of becoming better, is thus rendered much worse, to the accomplishment of their destruction." *Pref.* 2.

It is almost unnecessary to state, that Dr. Sintelaer was induced to publish his work entirely "in consideration of the frailty of mankind and the misfortunes that attend him," the main design of it being "no other than to convince the world that the cure of the venereal disease by mercury is, at the best, very uncertain, and often subject to great hazards and ill consequences, always to no small difficulties;—and that there are actually, in *rerum natura,* such remedies and antidotes as will free us of this enemy of our constitution, without the least aid of mercury." Dr. Sintelaer being thus so generous to others, has a fair right to shew a little kindness to himself. Accordingly he informs us that, without ostentation or vanity, he may safely affirm that his symptomatology, &c. of the disease is so exact and circumstantial, that nothing can possibly compare with it—

and therefore "it must needs meet with the approbation of the unbiassed and judicious reader."

There certainly is a great deal of curious matter in this same work of Dr. Sintelaer; but we can notice only a very few of his opinions or practices in this article. It appears that in his time the lytta was used internally for the prevention and cure of gonorrhœa, though our author does not seem to approve much of the medicine. In opposition to an anecdote related by a Dr. Greenfield, of a seaman who had kept himself free from gonorrhœa by tincture of cantharides, after having been dabbling in very troubled waters, Dr. Sintelaer asserts thus:—

> "I knew, some years ago, a young debauchee, who for twelve or eighteen months together and longer, made use by turns of all the whores he could pick up in the play-house, without receiving the least harm, but at last was most miserably peppered off by a substantial citizen's wife, when he least expected it." 31.

It is in the 4th, 5th, and 6th chapters of the second book that Sintelaer declaims on the poisonous effects of mercury, beginning with calomel. The only *fact*, however, which he brings forward against this luckless preparation is the following:—"Dr. Harvey tells us *a story* of an apothecary who gave three children a dose, each, of mercurius dulcis against the worms, and they all three died the same day." What the dose was, or how the mercurius dulcis was prepared, the deponent knoweth not.

On the other preparations of mercury many heavy curses and accusations are heaped by this ancient anti-mercurialist. From the anathemas against the mercurial ointment as a means of salivating, we shall extract the following passage, which might be taken as a text or motto for the modern declaimers against mercury.

> "It would be an endless piece of work to enter upon a particular recital of the mischiefs occasioned even by this kind of salivation, though reckoned, as I told you, the most benign, and the most successful of all the rest: what wandering and pungent pains and lameness it causes in the muscles and tendons; what tumours and inflammations in the glandulous parts; what spasms, tremblings, palsies, and convulsions in the nervous parts, and what insupportable pains, rottenness, and caries in the bones!" 180.

In the sixth chapter Dr. Sintelaer comes to issue with the "champions of Mercury," as he calls them, and here he lets out an observation of Harvey which seems to give him great uneasiness:—"*Mercury*," says Harvey, "*is only hurtful in a few, perhaps to one in five thousand.*" This assertion may not be unworthy the consideration of a few of the present generation, who convert the exceptions into the general rules, and thus broach doctrines and propose practices which are wild and injurious. The following extract will shew that Sintelaer anticipated all the modern writers on the mercurial disease.

"If you observe that after the cure of the pox by mercurial medicines, either some fresh pocky symptoms, such as did not appear before, but especially such as make their appearance in the glandulous and bony parts, as ulcers in the mouth and palate, or the roof of the mouth, and violent and continual pains in the bones; I say, if you find these symptoms appear after a cure of the pox by mercury, when nothing of it was observed before, or if you find these and other such like symptoms, which discovered themselves before the said cure become afterward more violent and frequent, you may then be fully convinced that they owe their origin chiefly to the malignity of the mercury, or at least to its intermixture with some slight remnants of the old pocky ferment; whence it is that we have given it the name of a mercurial or symptomatical pox." 221.

This explanation, Sintelaer observes—

"Leads us into the solution of a certain assertion maintained by several great physicians, both ancient and modern, viz. that the pox may lie lurking in our bodies for ten, twenty, nay thirty years;—which must be understood chiefly of the mercurial pox. For the mercurial poison will lie lurking for a long time in the body, till being separated and put in motion, it seizes upon the head and glandulous and bony parts, (unto which it is a constant enemy) and makes its appearance (after having lain dormant a great while) under such symptoms as have been mentioned before, which being the same that often attend an old pox, are consequently easily mistaken for that distemper." 222.

We have now adduced sufficient from Sintelaer to prove that there is little *new* in the present day, respecting the mercurial disease, and *pseudo-syphilis*.* The latter term is actually used by Sintelaer in the 2d chapter of the 3d book, which is headed—"Of the Mercurial or *Bastard-Pox*." We shall now proceed to the second new point, the *non-mercurial* treatment.

---

* Nearly 30 years before Sintelaer, viz. in 1683, Blegmy, a French writer, came to nearly the same conclusions as some of our modern practitioners, and adopted the same treatment that has been found successful by the army surgeons. He has one chapter "on the possibility of curing the venereal disease *without* mercury"—and particularly without salivation "flux de bouche." He speaks in so candid and rational a manner respecting the use and abuse of mercury, as to render his remarks peculiarly interesting at the present period.

As preliminaries, essential to the cure of syphilis without mercury, Sintelair enumerates different kinds of sweating. The first and most effectual, he observes, is by means of spirit of wine. And here Sintelaer anticipates Dr. Gower in this country, and an American practitioner, who is in a great rage with us for not attributing to him the original invention of this process. The following extract will set all claimants silent.

"In bed it is performed thus: the patient is laid quite naked in bed, and over his body are extended three or four half hoops, in order to keep the bed-clothes from touching his naked body; this done, you must place near the bed-side of the patient, a square tin box, with a small door on one side, and a tin pipe on the top, of such a length as to reach up to the bed-clothes, or rather to the heighth of the bedstead itself where the patient is laid. Things being thus prepared, you must put a small kettle or other vessel into the door of the before-said tin box, and put into it some spirit of wine, which being set on the fire, the vapour thereof will ascend through the pipe (one end of which is put under the bed-clothes) to the body of the patient and make it sweat most plentifully in a little time, which you may continue as long as you see it convenient; you must take care not to put in too much brandy or spirit of wine at a time, which would burn too fast, but do it gradually, and as often as you find the brandy consumed, supply it with fresh." 231.*

In regard to the means of curing the disease, they are precisely those which the army officers have lately employed for curing syphilis without mercury, to wit, low living, confinement within doors, open bowels, and diluent drinks—especially the compound decoction of sarsaparilla. Here Sintelaer introduces a letter from M. Pinket of Ghent to Dr. Blankard of London, describing a mode of curing recent venereal chancres, in nine days, as employed by the Spanish surgeons in the low countries about a century ago. This mode was to keep the patient warm in his bed, or at least in his chamber, on four ounces of meat in the 24 hours, drinking ad libitum the decoctum sarsæ compositum.† The

* Compare this description with Dr. Gower's Sudatorium, in page 649 of our first or quarterly series, vol. ii.

† Sintelaer made the patient drink four or five pints daily of the compound decoction of sarsaparilla, and every day forced the patient into a profuse perspiration, by means of the sudatorium above described. We have great reason to believe that practitioners of the present day often fail from not giving the sarsaparalla, whether in decoction, powder, or extract, in sufficiently large doses. We know a gentleman who has acquired great reputation by his cures—and they almost entirely depend on giving the abovementioned medicine in quantities four or five times greater than those usually exhibited. We recommend this hint to the attention of our brethren.

mercurial pox, as Sintelaer calls it, was treated in the same way. We shall introduce a single case, as a specimen of the many related by this anti-mercurial doctor.

"In July 1706, came to me the wife of a certain tent-maker, who having contracted the venereal disease gave me the following account of her present and past condition; she had, she said, as she believed, taken the advice of no less than forty practitioners, by some of whom she had been salivated all manner of ways, viz. by mercurial unctions almost all over her body, by excessive suffumigations, as likewise by Bolus's pills, powder, &c. and after having endured all these torments, miseries, and dangers, she was sent away with a pass for an incurable, and well she might be so, after so many violent and reiterated mercurial salivations, which had rendered the consequences much worse by this time than the disease itself was before.

"Some, it seems, among her doctors were so ingenuous and honest, as not to take any of her money; but others, who pretended to a more than ordinary skill and method in the cure of this distemper, had the good conscience to get out of her all they could, though their motto was, *no cure no money*, but their real design tended actually to nothing else, but, *all money all mercury*, as this poor creature had been sufficiently convinced to her cost.

"But, after all, she would perhaps have thought herself tolerably happy, had she not been reduced to such a condition by the mismanagement of some of her doctors as to be dismissed as incurable; her symptoms occasioned by the mercurial pox being such as made not only herself, but also some able practitioners to despair of a cure.

"For when she came to my house, her legs and arms were not only exceeding painful, and the bones thereof covered with hard nodes, but her head also, her throat, shoulders, and back full of deep and most putrid corroding ulcers; to add to her misery, she was already gone five months with child, which made her dread (as she had all the reason in the world to do) that the poor infant would partake, when born, of the miseries of its mother. In this distressed condition she had recourse to me for help, which I willingly afforded her; we began the cure after my usual method, with a specific purging powder, and after having ordered her such a diet, as I thought most suitable to her present disposition, she began to drink of the anti-venereal decoction, according to my direction, for thirty days successively, which had this good effect, that within the before-mentioned time all her ulcers healed up, without the use of any other remedies than the before-mentioned diet-drink, her nodes on the arms and legs disappeared, and she was not only restored to perfect health, but also, in due time, brought to bed of as lusty and well-look'd a child as could well be seen, without the least signs or appearance of any venereal symptoms or infection; and both the mother and the young infant continue ever since (as far as ever I could learn) in a perfect state of health." 305.

In concluding our notice of this book, we are, in the first place, under the necessity of pronouncing its author to be a QUACK. After all that is said about the amazing success of the *anti-venereal decoction*, the knave acknowledges that one of its component parts he must keep secret—and why? because, forsooth, if he divulged it, it would tend to his own detriment—"and to the no small encouragement of a sin, which is only too natural and too common already!" But while Sintelaer must be classed among Charlatans, it is evident that the modern doctrines respecting syphilis, pseudo-syphilis, and the non-mercurial treatment are but amplifications of what he had written more than a century ago. At the same time, it is abundantly evident that the mode of employing mercury at that time, by profuse and reiterated salivations, was the grand reason why so many injurious consequences resulted, and why other means were eagerly sought after for eradicating the disease. When mild and alterative courses of mercury were afterwards found so universally efficacious, and so seldom productive of mischief to the constitution, the medicine regained its claim as a specific, and the belief was gradually established that the disease could not be eradicated from the system without the mineral. How John Hunter, and the greater number of the profession, came to lay it down as a maxim, that chancres and other symptoms of syphilis went on increasing till the specific was applied, it is difficult to say, unless the disease has lately undergone some modification in its nature.* This maxim is now unquestionably proved to be erroneous; but we imagine that the superiority of the mild mercurial course, (where scrofula or other peculiarities of constitution do not forbid) over every other means hitherto devised for the cure of syphilis, will ultimately be established on a firmer basis than ever. It is not to be denied, that, partly from misapprehension, and partly from that love of novelty and change so inherent in the human mind, a great many practitioners, especially in the junior classes, have become unsettled in their principles of treating syphilis, in consequence of the recent investigations by the army medical officers and others. It certainly was not the design of those who conducted these meritorious investigations to produce any such effect as that alluded to; nor can the said investigations themselves, when viewed in their proper bearings, do otherwise than ultimately place the value of the mercurial treatment in a just point of

* Even Mr. Abernethy, in drawing a distinction between syphilis and pseudo-syphilis observes that—"the constitutional symptoms of the venereal disease are generally progressive, and *never* disappear, unless medicine be employed."

view, as they will render the practitioner less anxious to commence or push a mercurial course, where there is doubt respecting the true nature of the disease, or the ability of the patient's constitution to bear the remedy without injury. This appears, in a great measure, to be the opinion of the author, whose work is second on the list.

Mr. Bacot has had considerable experience in the treatment of Syphilitic complaints, and has issued his little work into the world with much modesty, and chiefly with the view of guiding his junior brethren in the use of mercury;

"By collecting and stating the evidence upon this subject; by drawing those conclusions which seem to be fairly deducible from the experiments that have of late excited so much interest in the medical world; and by endeavouring to restore mercury to that degree of confidence to which it seems fairly entitled, ascribing to it, at the same time, those just limits beyond which that confidence would be productive of mischief." 3.

Mr. Bacot introduces a neat sketch of the various modes of treating syphilis from the earliest records of its appearance in Europe. In this sketch we can notice but a very few passages. He remarks (p. 12) that Wiseman was in the constant habit of bleeding his patients previous to putting them on a mercurial course; and he thinks the practice might be revived, in many instances, with much advantage. He quotes the passage from Dr. Paris's pharmacologia, which we have extracted at page 85 of the first volume of this series, relative to the increased susceptibility of the system to mercury, after bleeding.

"Without any reference to the after exhibition of mercury, it not unfrequently happens, that a large and sloughy sore, attended with a high state of inflammation and acute pain, is rendered mild and tractable by a copious bleeding; it has often occurred to me, to see this effect produced by a spontaneous bleeding from the rupture of some vessel of the part: and this remark equally applies to some ulcers of the tonsils as well as to the same condition of buboes." 13.

This we have witnessed ourselves; but we have generally trusted to low living, confinement, warm clothing, and plenty of thin water gruel going to bed, for accelerating the mercurial impregnation, and shortening the period of cure. In respect to the late investigations and non-mercurial treatment, our author sums up his conclusions in the following candid manner.

"1. It is assumed, therefore, as an established fact, that all ulcers upon the parts of generation are curable without the use of mercury; but I cannot concede that, generally speaking, they are cured with equal celerity: they require more strict confinement, more attention to the state of the general health and to regimen, than is found necessary under a mercurial treatment carefully conducted; and, in some instances, the length of time requisite for their complete cicatrization is alone a serious evil. It may be also added, that under the non-mercurial mode of cure they frequently heal with hardened and elevated cicatrices.

"2. It must be admitted, that a great proportion of cases so treated are followed by a train of constitutional symptoms, which succeed to the cure of the primary sores in a period varying from six weeks to three or four months; and though medical men differ greatly as to the proportion of these cases, the causes of which difference it would not be difficult to explain, perhaps, if we take it at an eighth of the whole number, our estimate will not be far from the truth. The appearances thus produced are various; I might say, almost infinite, in degree at least. Some of these will be mentioned more particularly hereafter; but they have been confined, as far as my knowledge and experience go, to ulcerations of the tonsils, pains in the limbs, swellings of the joints, particularly the ancles and elbows, eruptions and ulcerations of various kinds upon the body, limbs, and in the hairy scalp, ophthalmia, great debility, and wasting of the flesh, occasional deafness, and, not unfrequently, pulmonary complaints. This latter circumstance has been adverted to by Mr. Rose; and, in adding my testimony to his, I am prepared to go somewhat farther, by expressing my belief that the debility and cachexy of habit produced by these constitutional symptoms, too often render the system prone to the attack of that formidable disease, the pulmonary consumption. Two or three such cases have fallen within my observation; and the subject is too important to permit me to pass it over without calling the attention of the profession particularly to it. In this mode a consumption may be established, in a habit predisposed to it, without, as formerly, admitting phthisis in the number of syphilitic symptoms. Thus, also, in the scrophulous diathesis, a node may be excited, which, so far from being venereal, will be most cruelly aggravated by the use of the specific.

"3. That these constitutional symptoms, when left to themselves, or treated with warm baths, antimonials, sarsaparilla, &c. have in a greater or less space of time been removed, and have not led to a diseased state of the membranes and bones, I also can testify; no such disease having occurred in my own hospital practice for these three years past, with the exception of one case; and this man had used mercury imperfectly and interruptedly for the cure of an eruption of the tubercular kind, and of a very doubtful character. It must however be admitted, that under this mode of proceeding the convalescence is often long and tedious, the symptoms apt to recur, and the health remains for a long time delicate and fluctuating." 31.

From all this our author thinks it apparent that either the natural history of the venereal disease has been mistaken from its first origin, or has become so modified in the course of time, as no longer to present the same phenomena as formerly. Mr. B. inclines to the *first* supposition. We confess that we are much more inclined to the *second*, since we imagine we perceive a considerable difference in the appearance and course of the disease at the present time, compared with what we remember of it thirty years ago. This idea of a modification, we think, is greatly strengthened by a circumstance stated by Mr. Bacot himself—namely, that no mention is made of bubo before the year 1540, though it is now one of the most common attendants on the primary symptoms. Leaving this discussion, however, and returning to practical points, we agree with Mr. Bacot in the following judicious counsel.

"Viewing the serious train of evils to which the non-mercurial practice tends, I would advocate the moderate and gentle use of mercury in all those cases of primary sore, where a mild mode of local and general treatment is productive of no beneficial change in the course of a reasonable period; at the same time being perfectly prepared to do without it in all those cases, and in those constitutions where its employment appears to be pernicious, knowing that I can dispense with its use without the dread of any of those more formidable consequences which formerly rendered the surgeon often a slave to the prejudices and fears of his patient or to his own; and being convinced that it is both much wiser and more safe to postpone its exhibition than to persist in its administration where the habit is irritable, and it appears to operate upon the system as a mineral poison only, calling into action that peculiar and anomalous class of symptoms usually called cachexia syphiloidea." 35.

Of the nature of the syphilitic virus we know nothing—of the modus operandi of mercury, little. Our author justly considers the cure of a chancre as incomplete, while any elevation or marked hardness about the cicatrix remains—consequently the remedy should be continued till this appearance is removed.

In respect to the best mode of administering the mercury, Mr. Bacot prefers the blue pill to all other preparations, combined with such a proportion of opium as will prevent its running off by the bowels. The mercurial inunction, he thinks, has no peculiar advantage, except where the irritability of the stomach is troublesome, or there is a wish to affect the system rapidly. "It then forms an admirable help to, or substitute for, the blue pill." Mr. Bacot concludes this introductory part of the work with judicious observations on the management of the patient during a mercurial course.

1. *Gonorrhœa.* Mr. Bacot believes that gonorrhœa is sometimes succeeded by constitutional symptoms; "but the proportion of these cases is very small, their character is peculiar, neither do they require mercury for their removal;—indeed it appears to be clearly contra-indicated." These constitutional symptoms, as Mr. Carmichael and others have shewn, are generally ushered in by pyrexia, the joints, especially the ankles, becoming swelled, red, and painful, with a minute papulous eruption about the shoulders,, arms, and back. The febrile symptoms are transient, and the whole usually give way to purgatives, antimonials, the warm bath, and confinement to bed.

2. *Chancre.* After all the labours of experienced and enlightened practitioners, the subject of primary sores on the genital organs has not been yet reduced to system. Mr. Carmichael indeed is of opinion, that one particular species of sore *only* is capable of producing the true secondary symptoms of lues. Mr. Bacot's experience will not permit him either to subscribe to this conclusion or adopt any specific classification of his own—at least he is quite sure "that many varieties of sore, independently of the sloughy chancre mentioned by Mr. Carmichael, lead to constitutional symptoms, differing in no respect from those he has described, and admitting of the same mode of cure."

Perhaps the earliest appearance of chancre is pustular, but, in a majority of cases, when they are presented to the surgeon, they have passed this stage, and exhibit ulcerating or sloughing surfaces varying in kind and degree almost *ad infinitum.* Even Mr. Hunter, as our author observes, admits that the character which he attaches to venereal chancre is *not* peculiar to it alone, since many sores that have no disposition to heal have, so far, the same character. Mr. Hunter describes a sore upon the prolabium, which he decides to be a chancre, *because*, independently of its appearance, there was a bubo in one of the submaxillary glands of the same side—thus making the presence of a bubo the proof of a venereal sore, and tacitly admitting the impossibility of recognizing it by the eye alone. In short, Mr. Hunter's definition of chancre is now applicable to but a very small proportion of venereal sores which are actually followed by constitutional affections. When we consider the several different textures composing the genital organs, and the great variety of constitutions, we need hardly be at a loss to conceive the variety of aspect which sores assume, even from the same virus. Without entering farther into these distinctions, Mr. Bacot undertakes to describe one or two peculiar ulcers, in which the exhibition of mercury would be unnecessary or improper; and, finally, enumerates a few distinct characters of ulceration, together with their consequences and treatment.

1. The first sore which he mentions is one described by Mr. Pearson, when speaking of cinchona in the treatment of lues:

"It is characterized by a great derangement of the general health, by a high state of inflammation of the part, by great local pain, and proceeds rapidly to the destruction of the parts. The situation of this sore is most commonly in the angle between the prepuce and glans penis; and those of a full habit of body, the young and the vigorous, are most liable to its attack. The most prompt and vigorous anti-phlogistic means are necessary to arrest the progress of this sore; and the blood taken away in these cases presents the usual inflammatory appearances, frequently in a very high degree. The exhibition of mercury, in this species of sore, is highly mischievous, and productive of the worst consequences; nor does it often happen, that secondary symptoms succeed in these instances. The destruction of the parts is so rapid, that the agency of the poison seems to be destroyed; and, as Mr. Pearson has remarked, mercury is perhaps not requisite for the after security of the constitution." 58.

The above observations apply only to the most aggravated forms. This sore is sometimes met with less violent in degree, and less rapid in its course. Here constitutional symptoms occasionally shew themselves in the shortest possible period, and are distinctly formed before either the system or the ulcer is reduced to a sufficiently quiescent state for the safe administration of the remedy, which, however, must be resorted to as soon as the condition of the ulcer will admit. These latter cases are of rare occurrence compared with the violent form before described. Two illustrative and interesting cases are detailed by Mr. B. in this place, which we recommend to the reader's attention.

2. Another ulcer of frequent occurrence is sometimes situated on the internal prepuce, sometimes on the glans.

"In both cases, the hardness surrounding the sore is very considerable; but, in the former situation, this hardness is very peculiarly marked: the surface of the sore is generally covered with a dark, liver-coloured slough, which falls off, and is succeeded rapidly by other sloughs, destroying the parts rather in depth than in breadth. The discharge from this sore is a thin, dark-coloured ichor; it is not attended with much pain, and is very much under the influence of mercury. The external application of this remedy, in the form of the black wash, is generally productive of very marked benefit." 63.

Although Mr. Bacot advocates the use of mercury in this particular sore, until the hardness of the surrounding parts is removed; still he is not certain that its most careful administration will secure the constitution against the attack of secondary symptoms. Notwithstanding the most carefully conducted mercurial course, our author has seen an eruption of copper-coloured spots, and an enlargement and excavated ulcer of the tonsils, and swellings of the elbow and ancle joints, succeed to the cure of this ulcer, in a period of from two to three months. "These symptoms are as much under the influence of mercury as the primary sore; they are neither tedious nor difficult to remove, and the flesh and strength are recruited as rapidly under the use of the remedy, as they had before rapidly declined." A case in illustration is given.

3. A third variety of sore, often met with and easily recognized, usually attacks the external prepuce or body of the penis, and generally appears to us, at first, as a dark coloured scab, spreading irregularly, and, on the crust falling off, leaving a surface rather elevated above the surrounding parts. The granulations are large, loose, and flabby; the edges hard, and of a deep red colour. "This sore calls for the exhibition of mercury, which materially facilitates the progress of healing." "When this remedy is omitted, constitutional symptoms succeed in a very large proportion; and I firmly believe, that the judicious use of mercury will most commonly prevent such an occurrence." In this ulcer there is such a tendency to the production of unhealthy granulations that escharotics are almost daily necessary.

4. What Mr. Howard calls the *aphthous* chancre belongs rather to a large family of sores, of which Mr. Bacot notices only two species—the one bearing a strong resemblance to the aphthæ of children, not acquiring any size, being numerous, and little sensible. These our author does not consider to be syphilitic. Another sore may also be called aphthous, inasmuch as it resembles the preceding, at the commencement, but soon spreads, preserving the circular form, acquiring a considerable size, and presenting to the eye an ashy-coloured unhealthy base, the edges of the sore, though not elevated, being often hard, and of a deep red colour.

"Here the early exhibition of mercury is in my opinion of the greatest use, though the cicatrization of this ulcer is often tedious under any mode of treatment, and it frequently leaves considerable induration of the part." 68.

In addition to these few specimens of ulcer, which our author has attempted to describe, "innumerable shades and varieties are to be met with, which baffle all description, and defy even the pencil of the artist." The judgment and penetration of the practitioner must, of course, be his guide through this labyrinth.

With the following sentiments of our author we entirely agree, being the practice which we have pursued, and the doctrine which we have preached, for very many years in this disease.

"The exhibition of mercury in the majority of primary sores, with the exceptions and restrictions I have mentioned, is, however, so safe and so generally beneficial, that where an ulcer continues for a certain time to pursue its course, and to resist all those mild methods of cure, both external and internal, which influence the progress of sores in other parts, I should not hesitate to have recourse to its exhibition: my object would be its mild, but gradual accumulation, not placing my patient, unless local circumstances rendered it necessary, under total confinement, and taking particular care that the affection of the gums does not advance too rapidly, or proceed to such an extent as to compel me to omit the remedy until I am satisfied that the cure has been effected. My endeavours would be directed to keep up a continued and unbroken action of the specific during the whole period, being convinced that the interrupted and careless use of the mineral too frequently leads to the establishment of anomalous symptoms, perplexing to the surgeon, and ruinous to the general health of the patient. I have said little of the local treatment of those sores in which the exhibition of mercury is determined upon, my intention not being to write a treatise on the disease; I shall, therefore, only observe, that they should be as little interfered with as possible, in order that the action of the remedy may be apparent upon them; the use of stimulant and escharotic applications being had recourse to when the state of the ulcer demands their employment." 70.

On the subject of buboes, Mr. Bacot makes many very judicious remarks and observations, for which we must refer to the volume itself.

5. *Constitutional Symptoms.* The endless combination of these, which every man of experience must have seen, renders it exceedingly difficult to do justice to the subject in a limited space. We can confirm the truth of the following sentiments.

"I would, however, premise one observation, which is this: that it is my firm conviction, that an attentive consideration of those cases of primary sore that come under our care, and a judicious, temperate, and cautious employment of mercury, will greatly narrow the circle of the constitutional symptoms, and render them of comparatively rare occurrence. In this opinion, I most heartily concur with Mr. Guthrie; but I must at the same time express my belief, that, although constitutional symptoms did not so often present themselves before the non-mercurial practice became general, when they did occur, they were much more serious; because, they were generally the result either of too profuse an exhibition of mercury, or of its improper action upon a constitution ill-disposed to receive it." P. 64.

The eruptive diseases attendant on syphilis, though almost infinite in number and occasional combination with other symptoms, may yet, Mr. B. thinks, be usefully classed under four distinct characters, viz. the papular, tubercular, copper-coloured, and pustular eruptions. These four families admit of subdivisions to an endless extent. Amongst the most common constitutional affections, the enlargement and ulceration of the tonsils must be numbered. A very frequent concomitant on syphilitic eruptions is a swelling of the joints—particularly the elbow and ankle joints, accompanied by severe nocturnal pain, and often external redness, much resembling rheumatism. Nocturnal pains in the head and limbs, though sometimes met with alone, are much more frequently precursors of eruptions. Between venereal ophthalmia and iritis it is not always easy to discriminate. During the existence of the above and various other constitutional symptoms, there is considerable debility, and wasting of the flesh. As for ozœna, node, rhagades, feci, condylomata, alopœcia, and several other formidable symptoms, our author is not acquainted with them "as the regular and natural consequences of syphilis." He will not assert that these diseases do not occasionally occur as consequences; but instead of considering them as the general rule, he is rather inclined to look on them as exceptions. The remark of Ferguson and Guthrie, that there are more demolished noses in Lisbon than in any other place of equal size, does not influence Mr. Bacot's opinion, as he considers this description of cases likely to occur in a large maritime city, visited by strangers from every part of the world—especially when it is considered how many of these people are likely from their mode of life, to have taken mercury improperly, and to have been exposed to vicissitudes of weather. In respect to a node, Mr. Bacot observes—

"But surely no one now would hazard a mercurial course upon the faith of such a symptom occurring three, five, or ten years, after the cure of a primary ulcer—a doctrine not long since implicitly believed, and acted upon almost universally." 89.

6. *Papular* eruptions are sometimes prominent, sometimes hardly elevated above the surface—they are often found in conjunction with ulcerated tonsils. The eruption of lichen is generally preceded by pains in the limbs, exacerbated by night, and not unfrequently there is pyrexia. In the *latter* case, Mr. Bacot thinks the complaint is not syphilitic, and he would be very unwilling to use mercury until the general health was amended.

"When the papular eruption makes its appearance from three to four months after the healing of a primary sore, and the flesh and strength have visibly and gradually declined, without any apparent derangement of the general health, there can be no doubt as to the propriety of using mercury; and it is astonishing how small a quantity will, in some instances, produce a most striking effect. With respect to the continuance of the course, the disappearance of the symptoms would be my principal guide; and it does not appear to me to be at all necessary to keep up the action of the remedy after this purpose has been fairly and entirely accomplished, nor can any stated time be fixed with precision for it discontinuance." 96.

7. Among the more remarkable varieties of the *tubercular* eruptions, is the small tubercle, which generally breaks out on the eye-brows, forehead, arms, or hairy scalp. It is early in its appearance. The crusts are irregular upon the surface, and leave ragged, unhealthy-looking ulcers when they fall off, which heal, however, without much difficulty. This cutaneous affection is often formed before the primary sore is healed; and it generally follows a small and superficial ulcer situated within the prepuce.

"The larger tubercle, usually denominated rupia, consists of a high and elevated scale; so peculiar that when once seen it can never be mistaken. The scab is generally large, and is most frequently met with on the arms, back, and head; the crust falling off leaves an ulcer with a glassy, shining, level surface, the granulations from which are unhealthy and loose: the health suffers considerably in general previous to the appearance of this symptom, and it is not unfrequently accompanied with an enlargement, and a ragged and superficial ulceration of the tonsils. I cannot trace this form of eruption to any particular character of primary sore, but it is one of the earliest forms of secondary symptom; and, notwithstanding what has been said to the contrary, I believe it to be strikingly benefited by the mild employment of mercury: the corrosive sublimate properly diluted, and applied in the form of a wash, exercises a powerful effect upon these ulcers." 98.

Mr. Harrison, in his second plate and fourth case, gives a good descriptien of this tubercular eruption. The subject was a young man, 22 years of age, of vigorous constitution, admitted into hospital in June 1819, the primary sore commencing as a pimple, which, in a fortnight, spread rapidly, in four or five days more acquiring a large size, and exhibiting a foul, sloughing, and irritable state, attended with large and frequent pulse, pain in the head, foul tongue, &c. He was bled largely, and had cooling aperient medicines, poultices and fomentations being applied to the sore, and leeches to the bubo. In the course of a few days the sore assumed a healthy appearance, and healed within the third week. The bubo suppurated, burst, and closed without ulceration, in six weeks. During the last fortnight, he took decoction of bark, but no mercury. He was discharged apparently in good health. Two months after his discharge, however, he returned to hospital, with a tubercular eruption (excellently represented in the plate, which we recommend to the profession) having a gentle and uniform elevation of the skin, of a crimson colour, feeling hard but not painful. When thrown off they exhibited foul ulcers. With these symptoms there were pains in the joints, and sore throat without ulceration. This patient was subjected to a course of mercury, which appeared to do good at first, but afterwards disagreed. It was then left off, and sarsaparilla employed, under the use of which the sores healed, and the health and strength were soon regained.

Of the pustular eruptions Mr. Bacot has little experience; but he is induced to think they are not really syphilitic.

"Blotches," says Mr. Bacot, "and spots of a copper-colour, some of which are covered with a light bran-like scurf, are to be seen in every variety of form and degree, either alone or in combination with sore throat and swellings of the joints: here, as in the former cases, I am inclined to make a great distinction, when I find the general health much deranged, the tongue loaded and furred, and the appetite gone: under these circumstances, I should always withhold the exhibition of mercury, until, by proper evacuations and attention to the general health, I had given my patient the benefit of a delay, which will, in many instances, render all farther medical treatment unnecessary." 99.

True it is that, whatever plan may be pursued, these eruptive phenomena will eventually disappear, but where they continue to linger for a long time, and are attended with their usual accompaniment, languor, debility, and disturbed rest, Mr. Bacot cannot understand the advantage of delaying that remedy which repeated experience has taught him to rely on with confidence, in removing the symptoms rapidly, while "under its judicious use, the strength, the flesh, and the animal spirits will visibly return." A case is here related, shewing how tedious the return to health occasionally becomes, where these cutaneous affections are permitted to run their course. We fully agree with our judicious author in the following sentiments:—

"In combination with mercury many medicines may be usefully joined; and of these I think it difficult to speak too highly in praise of sarsaparilla; like mercury, it has had a variety of fortune: at one time it has been exalted and praised with a degree of hyperbole; at another period, it had lost its credit with the profession entirely; at present, its virtues appear to be more rationally estimated, and, when genuine, (for its adulteration is both frequent and of difficult detection,) it exerts a very beneficial influence in many anomalous cases, where mercury alone appears to disagree; and in conjunction with small doses of that mineral the effect appears to be more decisive than could be produced by the employment of either medicine singly." 103.

Mr. Bacot's little work concludes with some judicious observations on sore throat and ophthalmia, which deserve the attention of the practitioner in particular. Mr. B. notices a large ulcer of the tonsils, with ragged and unequal edges, surrounded by a deep coloured inflammation, extending over the velum pendulum and uvula, preceded and accompanied by much febrile action and uneasiness in swallowing, when *hot substances* are taken into the throat. Brisk purgatives, free blood-letting, and cooling diet soon induce a rapid change in this state of things. The large excavated ulcer of the tonsils, however, attended by cutaneous eruption, void of pain, and not surrounded by inflammation, though it may be kept in check by sarsaparilla, the mineral acids, &c. is quickly and permanently cured by mercury. "If it be thought desirable to put an immediate stop to the progress of the ulceration, the corrosive sublimate, in the form of a gargle, is a most efficacious and powerful remedy."

Ophthalmia, a secondary affection, is rarely met with as a solitary symptom. It is often attendant on the eruption of simple lichen, and imperatively demands the early use of mercury, in addition to the common means employed for the reduction of ophthalmic inflammation. Near the conclusion of the work Mr. Bacot observes, that, although he has not enjoined total confinement during the administration of mercury, yet, upon the whole, in this variable climate, seclusion is desirable, "because the legitimate effect of the specific upon the habit requires to be watched with great care, and the case is likely to become complicated, and to embarrass the practitioner, where cold is taken, or any other diseased action arises while the system is under the influence of mercury." We cannot, indeed, too strongly impress on the minds of young surgeons the necessity of confining their patients, if possible, during the administration of mercury. We have so often seen the good effects of attending to this rule, and the miserable consequences of neglecting it, that we cannot avoid taking all opportunities of holding up the good and evil to view, as an inducement to the one, and a warning against the other.

We must conclude our analytical notices of Mr. Bacot's little work, by recommending it to the surgeon, and especially to the young surgeon, as containing judicious advice and safe rules of conduct, where the practice is unsettled, and the indications doubtful.

The manuscript work, forming the third article at the head of our list, is a very erudite research into the origin of syphilis, and an elaborate disquisition on the use and abuse of mercury, not only in venereal, but in many other diseases. We are sorry that the nature of the investigation and the narrow limits of a review preclude all hope of giving any thing like a satisfactory analysis of the production in question—and we regret this the more, as we believe Dr. Thomson has no intention of committing the manuscript to the press.

The investigation into the origin of syphilitic diseases forms the concluding part of Dr. T's MS. though we think it should have been placed in front, and on that acoount shall commence with it here.

Our author's creed is *non-importation*. The controversies, he thinks, which have arisen respecting the origin of syphilis, may be traced to the intimate connexion it has with moral character. One nation has been anxious to throw off the odium on another—generally on its political rival:—"Ab Italia *Gallicus* affectus vocatur;—a gallis autem morbus *Neapolitanus*." All, however, have pretty generally agreed to make America (which had not then such sturdy champions to defend her character) the fons et origo mali. Dr. Thomson does not think the investigation into the origin of syphilis a mere matter of curiosity, nor can he agree with the sentiment of Celsus—"non intersit quid morbum faciat sed quid tollat." He thinks it a very important question to determine whether the disease proceeds from a specific poison transmitted from generation to generation, through more than three centuries—or has been thousands of times generated *de novo* by impure sexual intercourse, and thus may have existed, more or less, in all ages. This last idea would deprive the disease of many of its imagined horrors, and would account for the facility with which it is now eradicated, even without the assistance of medicine. We have ever considered the story of Columbus's sailors being the vehicles of this disease from America as completely fabulous; and therefore we agree with Dr. Thomson's sentiments, as shewn in the following extract:—

"Indeed the whole of the received account is extremely improbable, that *sailors*, after a long and successful voyage, landing on the northern coast of Spain, objects of curiosity, ready to embark again to reap the fruits of their discoveries, and the wealth the new countries were supposed to abound with, should have been sent off to act as *soldiers* at the siege of Naples, labouring under a new and horrid disease which must have been of some months' duration, and have incapacitated them from every kind of exertion; but that neither Columbus himself or his brother, who left such accurate narratives of his voyage from his MSS. should not make the least mention of such disease being discovered among the natives, or prevailing among the crews of their vessels, is certainly still more difficult to be reconciled with reason, and afford strong presumption of error." P. 42.

One thing is certain, that whether of foreign or indigenous origin, the disease is not now what it was—and in all probability varied in every age and country. It had changed in Boerhaave's time, as the following extract will shew. He says—

"*Primo* incipit a pustulis *genere diversis* in genitalibus, licet interdum, sed raro, in capite. *Secundo*, pustulis varis similibus, hinc Spansche Pokken. *Tertio*, aliæ planæ, minimæque extantes, *hæ* non sunt nostris temporibus. Aliæ colore subalbido, e quibus squammæ resolvebantur, et caro sub his cancrosa apparebat. Hæ sunt quæ hunc morbum ab aliis distinguunt." *Boerhaave.*

That erroneous ideas might have prevailed in former times is not to be wondered at, when we see that in our own time John Hunter, and thousands after him, gave mercury in every case of chancre, on the assumption that without it the sore would pursue a destructive and progressive course. We need not say that this notion is proved to be utterly void of foundation in fact.

It appears that Gabriel Fallopius, who read lectures on the "*Morbus Gallicus*," sixty years after its supposed importation, is the first who mentions its foreign origin; for Nicholas Massa, who preceded him, and to whose writings Fallopius added very little, did not suspect it. Boerhaave, who is said to have "examined systems by experiments, and formed experiments into systems," acknowledges that Massa wrote thirty years after the appearance of this disease, with so much candour as to bear the palm from all who went before him; yet Massa's account of the disease, as given by Boerhaave, will not apply at all to the disease as it now exists, or indeed as it existed in Boerhaave's own time. It is extremely probable indeed that, as the disease became older, it spent its force, for Astruc, Fracastorius, and others, observed that its symptoms were milder in their days.

But while the foreign origin of syphilis was doubted, even in the earliest periods of its history, all seem agreed that a new and terrible disease broke out about the dispersion of the army besieging Naples. And as a state of doubt is painful to the human mind, the explanation offered of a foreign origin was well calculated to satisfy the prejudices of European nations, while it served the physicians with an apology for their want of success in curing the disease. We cannot wonder at the rapid manner in which the disease spread, however first engendered, nor at the alarm excited in the mind of Governments and people, when we reflect that it was communicated by lying in the same bed, by the clothes, gloves, or money of the patient. The breath was also thought to be infectious, and we find Cardinal Wolsey indicted for whispering in the king's ear, while supposed to be labouring under the venereal disease. Who could suppose them speaking of syphilis as now seen among us? Dr. Thomson thinks that the circumstances attending the army of Charles VIII. were very favourable to the production of the disease; the said army having lain long before Naples, in want of provisions, and in a state well calculated for the generation of infection.

Our author thinks that evidences of the existence of syphilitic complaints may be found in the writings of the ancients, and the records of the Chinese, though these diseases, from certain combinations of causes, have been more virulent and prevalent at one time than at another. All the ancient books of the Chinese which make mention of syphilis, describe it not as a new but an old disease—" venenum actionum turpium." Celsus describes no less than nine different species of ulcers on the genitals, and how can we account for these, except as consequences of impure sexual intercourse? What, Dr. T. asks, can he mean by " cancrum qui in cole nascitur," unless it be chancre?—Perhaps a more particular description would have been given, had the Latin language not been more chaste than the Greek, as Celsus observes.

" That the various symptoms of syphilis might exist without their receiving a distinguishing name, is no more calculated to surprize us than the fact that small-pox and measles were for ages regarded the same disease, and only distinguished in modern times; not to mention the chicken-pock, confounded with both till separated by Heberden. Rhases, says Galen, was well acquainted with small-pox, which prevailed in his time, and many have thought it described by Hippocrates. Such has been the confusion on these subjects. 53.

We have another striking instance of inconsistency, error, and contradiction in the subject of gonorrhœa, which is said not to have been known in Europe till fifty years after the discovery of America, in contradiction to positive facts shewing its existence long before, as the laws respecting stews, and the "perilous disease of brenning," fully testify. But we must leave the origin of syphilis as we found it—

"For shadows, clouds, and darkness rest upon it."

We come now to the subject of mercury as a remedy in syphilis and many other disorders. Dr. Thomson has had ample experience of this medicine, so much lauded and condemned by different writers, in various climates, and on various subjects, and so far his testimony is entitled to much respect.

Dr. Thomson cannot ascribe to mercury any thing like a *specific* character, in the cure of syphilis or any other disease; but acknowledges that it is the best remedy hitherto discovered for syphilis, when judiciously administered, and provided there is no ideosyncrasy of constitution to contra-indicate its employment. This is the pith of Dr. Thomson's argument, but he brings forward a great deal of curious and important matter in proof or illustration; of which, however, our narrowing limits will permit us to make very little use. We have shewn, in many parts of this article, that the opinion is not new, respecting the effects of mercury in sometimes aggravating instead of curing venereal diseases. Boerhaave himself, speaking of this "horrenda labes," says it was "nunquam autem magis noxia quam si *mercurio* infecti hominis humores virulentos dissoluti sint."

"Many revolutions," says Dr. T. "have taken place in the use of mercury during the last three centuries, owing to the rashness of some and the timidity of others; and it is curious to observe, in the perusal of the older authors, how the great rival nations have alternately rejected and had recourse to mercury. When decried in England, it has been extolled in France; and when it lost its credit in France, it was again resorted to in England. These changes have taken place several times, and indeed the methods of cure adopted in the two countries at the present hour are in illustration of the fact; for while *we* are endeavouring to prove the inutility, if not indeed the danger of mercurial remedies, it comes within my own knowledge, in some of the first public establishments in France, that one preparation alone, and that the most powerful, is relied on almost universally for the cure of venereal disorders." 4.

Hunter himself, who says that nothing can shew the unsettled and ungrateful mind of man more than his treatment of this remedy, acknowledges that it often produced *nodes*, which he considered of a scrofulous nature, and pains resembling those of rheumatism. In short, there can be no doubt that the symptoms caused by mercury were too often confounded with those of the disease for which it was administered, and thus very dreadful effects produced by the error. We cannot therefore be surprized at the frequent passages in the old authors, proving that they often regarded the remedy as worse than the disease.

> "Graviora morbis patimur remedia,

> "Nec tanta vita est vivere, ut possis mori:"

> "Irrita letiferos auxit medicina dolores

> "Crevit et humana morbus, ab arte meus;"

are specimens of the exclamations and epitaphs of sufferers from mercury.

Dr. Thomson very properly censures John Hunter for asserting that the cure of syphilitic affections would go on equally well whether the patient drank a bottle of wine daily, or kept to barley water. We have seen this plan pursued, and Hunter's authority quoted, but the cure was never so certain, speedy, or easy, as where abstinence and confinement were enjoined. Dr. Thomson's sentiments respecting mercury in syphilis may be gathered from the following extract.

"For my own part, I will neither add to the eulogiums which have so lavishly been bestowed on mercury, nor range myself with those who, shocked at the consequences of its abuses, would engender a prejudice against its use, and deprive us not only of a safe and effectual remedy for syphilitic disorders generally, but, by exaggerating its morbid effects, discourage its use in other diseases. It surely can be no reason because the virtues of a remedy have been abused or overrated by our predecessors, that we should, by raising a clamor against it, cause it to sink as far below its first level as I am willing to allow it has at times stood above." 8.

We every year see this the case with other remedies which, being too highly extolled by their introducers, as applicable to numerous diseases, have sunk into unmerited neglect, as good for nothing. We may probably be justified in predicting, however, that mercury will not be exploded in the cure of syphilis, unless that disease change greatly from what it is at present; because the same ground which has been lately taken, has been trodden over and over again, and still men have returned to the remedy and considered it the best we possess. In these and all other medical discussions, where so much uncertainty, so much fallacy abounds, we should follow the advice of Lord Bolenbroke—"by reasoning cautiously, pronouncing modestly, practising sincerely, and hoping humbly." That observation of Boerhaave, who acknowledged that he knew of no remedy but what became so by its proper use—"nullum se cognovisse remedium quin solo tempestivo usu tale fieret," is peculiarly applicable to the exhibition of mercury. In the hands of a judicious practitioner it will fulfil a very considerable number of different, and apparently contrary indications—in the hands of ignorance, temerity, or indiscrimination, it will often do more harm than good. But while, with Dr. Thomson, we believe that a carfully conducted mercurial course is the surest and quickest cure of syphilis, we heartily join with our author in expressing our unlimited admiration of those who have, by cautious and ample experience, proved the power of Nature over the venereal virus, when assisted by rest, low diet, and antimonials. The army medical officers are, on this account, entitled to the gratitude of the profession, for having let in a flood of light upon the nature of syphilitic diseases.

In tracing the pernicious effects of mercury on the human constitution, when taken in excess, Dr. Thomson alludes to the circumstance of men escaping with *whole bones*, who have taken great quantities of mercury in tropical or other climates for acute or chronic diseases; and here, we must say, that Dr. Thomson makes use of very lame arguments, and defective reasonings. Dr. T. has served in hot climates, and he acknowledges that the affections of the bones attributed to mercury "very seldom take place;" but that they *never* happen, "he is *convinced* is not the case." Now to this we would reply in the words of the lawyer in the play —"no *convictions*, sir;—stick to *facts*." Dr. Thomson does not produce a single fact, nor does he assert that he ever saw a case of the kind. He is *convinced*, however, that they do occur—because "it is consonant to *reason* that they should." Again, say we—"no reasonings—stick to facts." For our own parts, we have seen, at least some thousands, who had undergone courses of mercury for dysentery, hepatitis, fevers, &c. and we cannot charge our memories with a single instance where diseases of the bones were the consequence. Dr. Thomsom, indeed, endeavours to reconcile this exemption with the doctrines of the day, by *supposing* that the effects of mercury taken in foreign climes do not become apparent till the return of the patients to northern latitudes. But as this is, at the best, but a *petitio principii*, we shall pass it over. The conviction on our own minds is, that it is only in syphilitic diseases—or, at all events, that is *prin-*

*cipally* in syphilitic diseases, where the exhibition of mercury is followed by affections of the bones—but whether from the disease or the remedy, we do not know, nor do we think that there is, as yet, clear and unequivocal evidence on the subject.

In respect to the curative powers of mercury in the ardent fevers of tropical climates, especially the West Indies, our author coincides with the most experienced practitioners, that, although where ptyalism takes place, safety is generally insured, yet that the mercurial action can seldom be set up in time, where the fever runs so high as in the concentrated endemic of the western world. We should not therefore, he properly concludes, trust to mercury, but rather to blood-letting and other antiphlogistic measures for the reduction of high febrile excitement, calling in mercury as an adjuvant for the restoration of impeded or deranged secretions in the system. While detailing some interesting facts relative to the fever which scourged our garrisons in Guadaloup, in the year 1810, Dr. Thomson relates his own case, where a single detraction of 56 ounces of blood, from a large orifice, checked the disease in embryo, and was succeeded by sound refreshing sleep, with a kindly determination to the surface, which, with some aperient medicine, perfected the cure.

From fever the transition is natural to dysentery, a disease second, if not equal, in importance to fever, both in respect to the mortality and the prevalence of the complaint in hot and unhealthy climes. Dr. Thomson appears to have appreciated the utility of bleeding, mercury, and opium, in dysentery. Although mercury has been more generally prescribed in the chronic than in the acute forms of this disease; yet, says Dr. T. "there is no fact in medicine, of which I am more convinced than that mercury possesses, in a high degree, the power of relieving inflammation of the intestines and diminishing their mucous discharges."

> "The increased action," says he "of the salivary glands relieves the intestines, for I have observed, almost uniformly, in a vast number of cases that, as soon as the mouth became affected, the bowels were relieved; and this fact was so well established at last in our hospitals, that the men looked forward to it with confidence, for diminution of pain and discharge of blood." 34.

We can confirm Dr. Thomson's observations by personal experience of their correctness. We are gratified also in finding our experience confirmed by so judicious a physician as our present author, in the following important point of practice in dysentery.

"The mode of administering mercury, in which I chiefly relied, was that of combining calomel with opium. In this form of prescription we safely avail ourselves of the ease opium affords. Huxham observed, that purgatives procured a truce which opium prolonged, but that this often cost dear. The *relief* was temporary, but the symptoms returned with increased violence. This is not the case when it is combined with *calomel*, to which it is indeed a powerful auxiliary, tending to assist and accelerate its effects." 35.

The lancet and aperients, where inflammatory symptoms run high, must, of course, precede or accompany the administration of mercury in dysentery.

Our limits have prevented us from giving more than the most imperfect outline of Dr. Thomson's manuscript, which is characterized by diligent research, sound judgment, and liberality of sentiment.

We have only now to notice Dr. Swediaur's new edition, for the purpose of recommending it as a class-book for the young practitioner, without which he will have a very imperfect idea of the history, symptoms, varieties, and treatment of syphilis and syphilitic diseases.

Dr. Swediaur is a veteran, now retired, *in otio cum dignitate*, we believe, in Paris. He had spent many years in England, where he published the first edition, as far back as 1784, at Edinburgh. Two years afterwards he published a second edition in London, without change of text. In 1788, a third edition, with corrections and additions, was given to the public, after which period Dr. Swediaur pursued, without intermission, his researches and observations on the subject, till he amassed a great collection of materials, which induced him to publish a French edition at Paris, in 1798, being almost a new work. After this, he published two other editions, viz. in 1801 and 1809, with great additions and improvements. In the present English edition our learned and indefatigable author has developed his subject as far as the present state of our knowledge (with the exception of the recent experiments on the non-mercurial practice) would permit—omitting nothing essential that could elucidate the nature or treatment of this important class of diseases—not even the personal sufferings of himself, while labouring under them.

In the first chapter our author developes the history of diseases of the genitals, known to the ancients previously to the appearance of syphilis in Europe. In the second chapter he endeavours to explain and elucidate the history of the disease, *demonstrating* we had almost said, "the falsity of the opinion of those who maintain that it came to us from America by means of the Spaniards." In this opinion he is joined by the learned Sprengel, and others of minor note. He has rendered the opinion very probable that the syphilitic disease began to appear in Europe about the year 1483—at least he adduces evidence that it had spread in Italy and Germany, before the return of Columbus from his first voyage to America.

"I have proved that it showed itself in the commencement of its appearance in Europe, as an epidemic disorder, very contagious not only by contact with infected bodies, but also by their clothes and their utensils, and probably even by the atmosphere, without any species of contact; that there died of it a great number of individuals, and that it was regarded for this reason as pestilential; that it had then a great resemblance to elephantiasis, and especially to the *yaws* or *pian* of the Africans; that it lost by degrees the character of a pestilential and epidemic cutaneous disorder, and that it has ended by becoming mild, as we find it at the present day, and by its being communicated with much less facility." *Pref.* ix.

In the third and following chapters, our learned author treats of the effects of the syphilitic virus on the genital organs. He has made gonorrhœa a principal object of his researches. He considers the real primary seat of the disorder in men, as always in the cavity of the urethra, at the *fossa navicularis*, directly under the frenum. When it is found seated lower down in the urethra, it is generally, he thinks, the consequence of improper treatment, or some fault of the patient.

He thinks that both those who advocate and those who deny the identy of the syphilitic and gonorrhæal virus, are occasionally wrong in generalizing too much, and in speaking so positively and so lightly on a point of so much importance to physician and patient.

"I think I have proved to demonstration in the third chapter, that the blennorrhagia of the genitals of the two sexes owes its origin sometimes to the venereal or syphilitic virus, properly so called, and sometimes to some other acrimony applied to the urethra or the vagina. I have there related several well-proved facts, which demonstrate that this discharge is often really venereal or produced by the syphilitic virus, amongst others a case of my own, in which syphilis was the effect and evident consequence of a gonorrhœa. I have observed a great number of similar cases, in which syphilis was the consequence of a neglected or ill-treated gonorrhœa. On the other hand, I have established by well averred facts, that the blennorrhagia of the genitals is often evidently very different in its origin and nature from that produced by the syphilitic virus. It will easily be conceived of what importance this distinction is in practice; where on the one hand we see practitioners treat all gonorrhœas as venereal with mercurials, and on the other, by an ill-founded theory they suffer the syphilitic virus to be communicated and the disorder propagated through whole families, without giving themselves any trouble as to the unfortunate results." xii.

In the chapter on chancres or ulcers of the genitals, Dr. Swediaur endeavours to establish "essential distinctions" between the truly and false syphilitic sores, in order that the treatment may be discriminated. Our author considers the non-mercurial practice lately tried in England as "far from being established on any solid foundation." At the same time, he thinks it very imprudent to exhibit mercury indiscriminately in all venereal sores—and particularly to exhibit the oxymuriate of mercury as they do in France, when milder preparations are sufficient. In chapters 12 and 13 of vol 1, our author shews that "primary, *i. e.* recent local syphilitic ulcers and buboes, are generally radically cured in three or four weeks, by simple external and topical applications of mercury." In our opinion, this passage speaks in favour of the non-mercurial practice—at least we think it proves the possibility of curing syphilitic sores without mercury—for the external or topical application of that remedy to ulcers, we believe, is next to nothing, as a mean of permanent cure. But while our author objects to trusting to sarsaparilla and the non-mercurial treatment, in the moist and cold climates of Europe, he thinks the sarsaparilla might be usefully employed as an auxiliary, and to secure the constitution against the effects both of the poison and the remedy.

In the French edition of 1798, Dr. Swediaur introduced a chapter on the "*mercurial disease*," which subject, he says, was then quite new, as he could not derive any information from books. "The authors who have since written on the subject, says Dr. S. have copied what suited them from my work, without acknowledging it, but have added nothing to the information I had already given respecting the disorder, or the mode of treatment I had pointed out."

In the treatment of mercurial diseases, Dr. Swediaur recommends the sulphurous mineral waters, and, in obstinate cases, the sulphurous vapour baths. Our author introduces a chapter, (the fifteenth,) containing a history of the syphilitic disease which showed itself, at the close of the last century, in Canada; which disease, Dr. S. thinks, throws new light on the history of syphilis, and on the action of the virus. The account of this disease which was rendered to the English Government, at the time, by an enlightened physician, has never been made public, but was obtained by him, and faithfully abstracted in the chapter abovementioned.

In fine, we cannot close this notice of Dr. Swediaur's new edition, without pressing it on the attention of the rising generation of the profession, as a class-book on the subject of syphilis, more copious, complete, and judicious, than any work of the kind in the English language.

