# Errata

**Published:** 1822-03-01

**Authors:** 


No. 7, page 598, *for* Henry, *read* John Thomson.

